# Supplementary material for: GenPipes: an open-source framework for distributed and scalable genomic analyses
Source: Gigascience. 2019 Jun 11;8(6):giz037. doi: 10.1093/gigascience/giz037 (PMC6559338; doi:10.1093/gigascience/giz037)

Figure S1

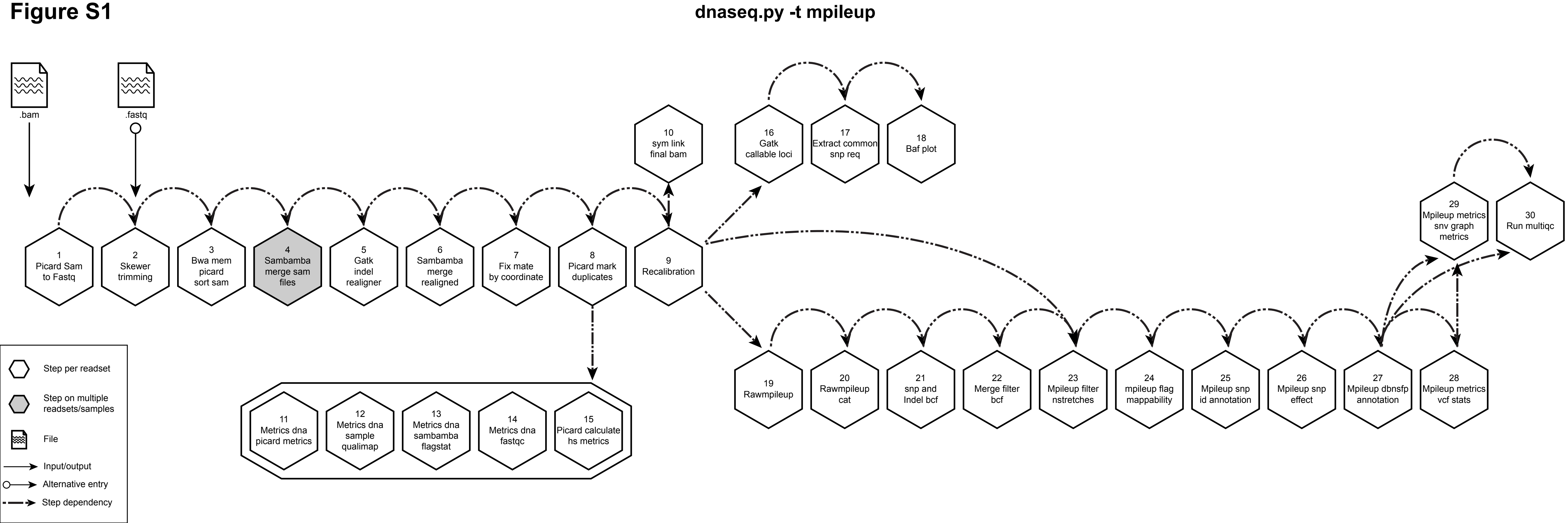
dnaseq.py -t mpileup
.bam
.fastq
1 Picard Sam to Fastq
2 Skewer trimming
3 Bwa mem picard sort sam
4 Sambamba merge sam files
5 Gatk indel realigner
6 Sambamba merge realigned
7 Fix mate by coordinate
8 Picard mark duplicates
9 Recalibration
10 sym link final bam
11 Metrics dna picard metrics
12 Metrics dna sample qualimap
13 Metrics dna sambamba flagstat
14 Metrics dna fastqc
15 Picard calculate hs metrics
16 Gatk callable loci
17 Extract common snp req
18 Baf plot
19 Rawmpileup
20 Rawmpileup cat
21 snp and Indel bcf
22 Merge filter bcf
23 Mpileup filter nstretches
24 mpileup flag mappability
25 Mpileup snp id annotation
26 Mpileup snp effect
27 Mpileup dbnsfp annotation
28 Mpileup metrics vcf stats
29 Mpileup metrics snv graph metrics
30 Run multiqc
Step per readset
Step on multiple readsets/samples
File
Input/output
Alternative entry
Step dependency

Figure S2

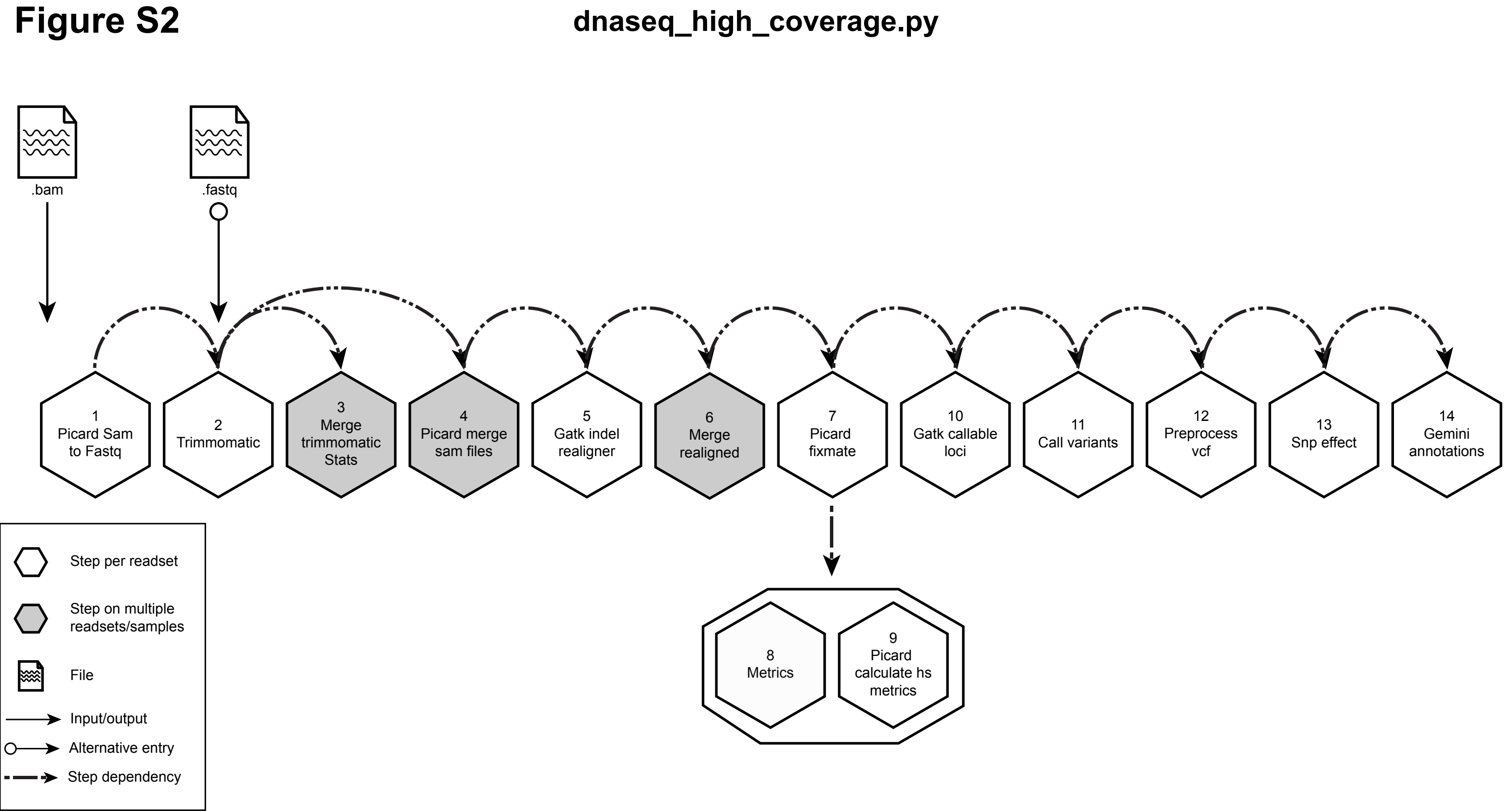
dnaseq_high_coverage.py
.bam
.fastq
1
Picard Sam to Fastq
2
Trimmomatic
3
Merge trimmomatic Stats
4
Picard merge sam files
5
Gatk indel realigner
6
Merge realigned
7
Picard fixmate
10
Gatk callable loci
11
Call variants
12
Preprocess vcf
13
Snp effect
14
Gemini annotations
8
Metrics
9
Picard calculate hs metrics
Step per readset
Step on multiple readsets/samples
File
Input/output
Alternative entry
Step dependency

Figure S3

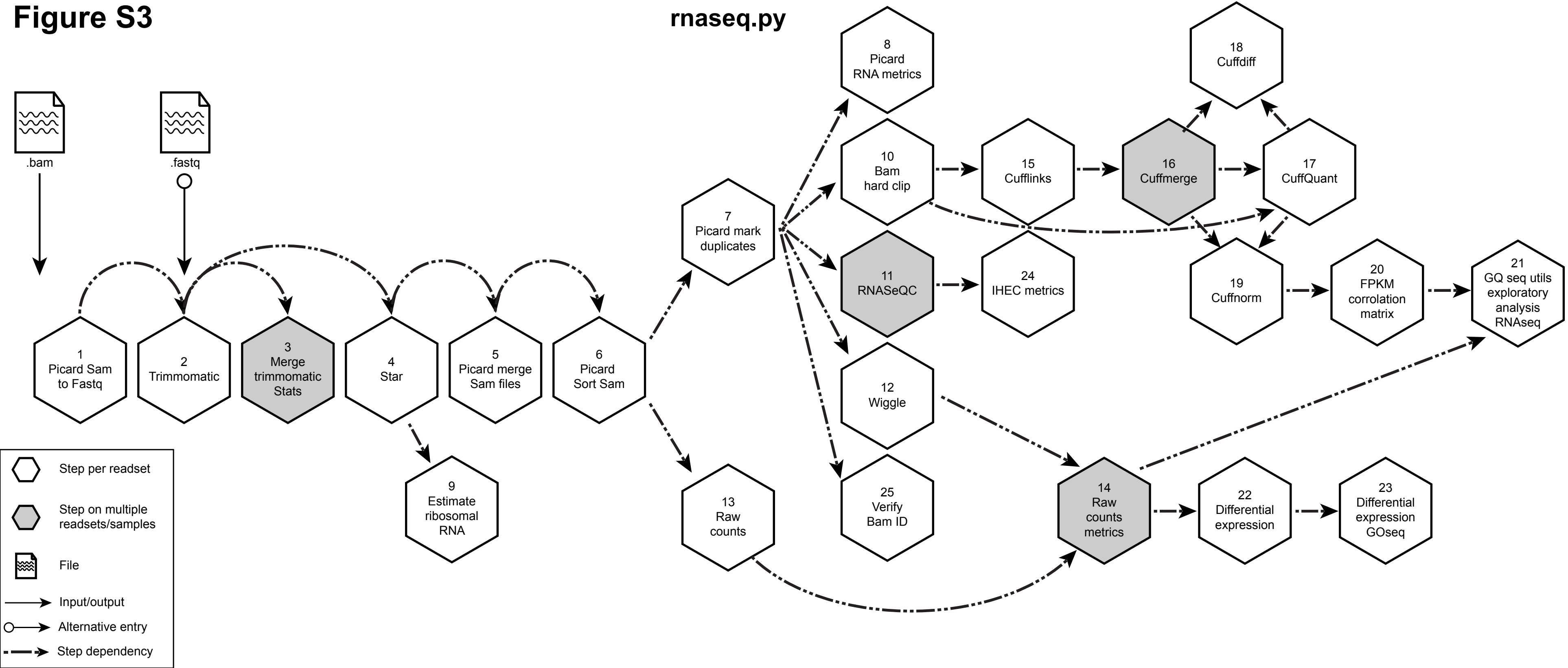
rnaseq.py
.bam
.fastq
1 Picard Sam to Fastq
2 Trimmomatic
3 Merge trimmomatic Stats
4 Star
5 Picard merge Sam files
6 Picard Sort Sam
7 Picard mark duplicates
8 Picard RNA metrics
9 Estimate ribosomal RNA
10 Bam hard clip
11 RNASeQC
12 Wiggle
13 Raw counts
14 Raw counts metrics
15 Cufflinks
16 Cuffmerge
17 CuffQuant
18 Cuffdiff
19 Cuffnorm
20 FPKM corrolation matrix
21 GQ seq utils exploratory analysis RNAseq
22 Differential expression
23 Differential expression GOseq
24 IHEC metrics
25 Verify Bam ID
Step per readset
Step on multiple readsets/samples
File
Input/output
Alternative entry
Step dependency

Figure S4

rnaseq_light.py

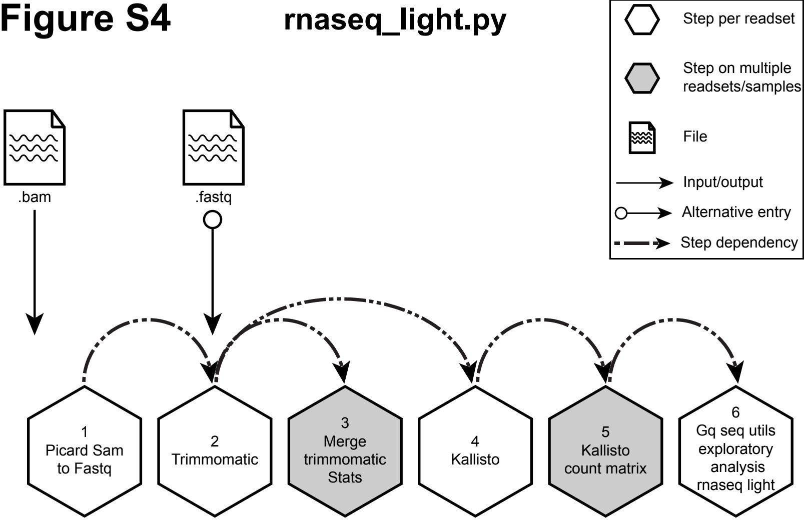

Figure S5

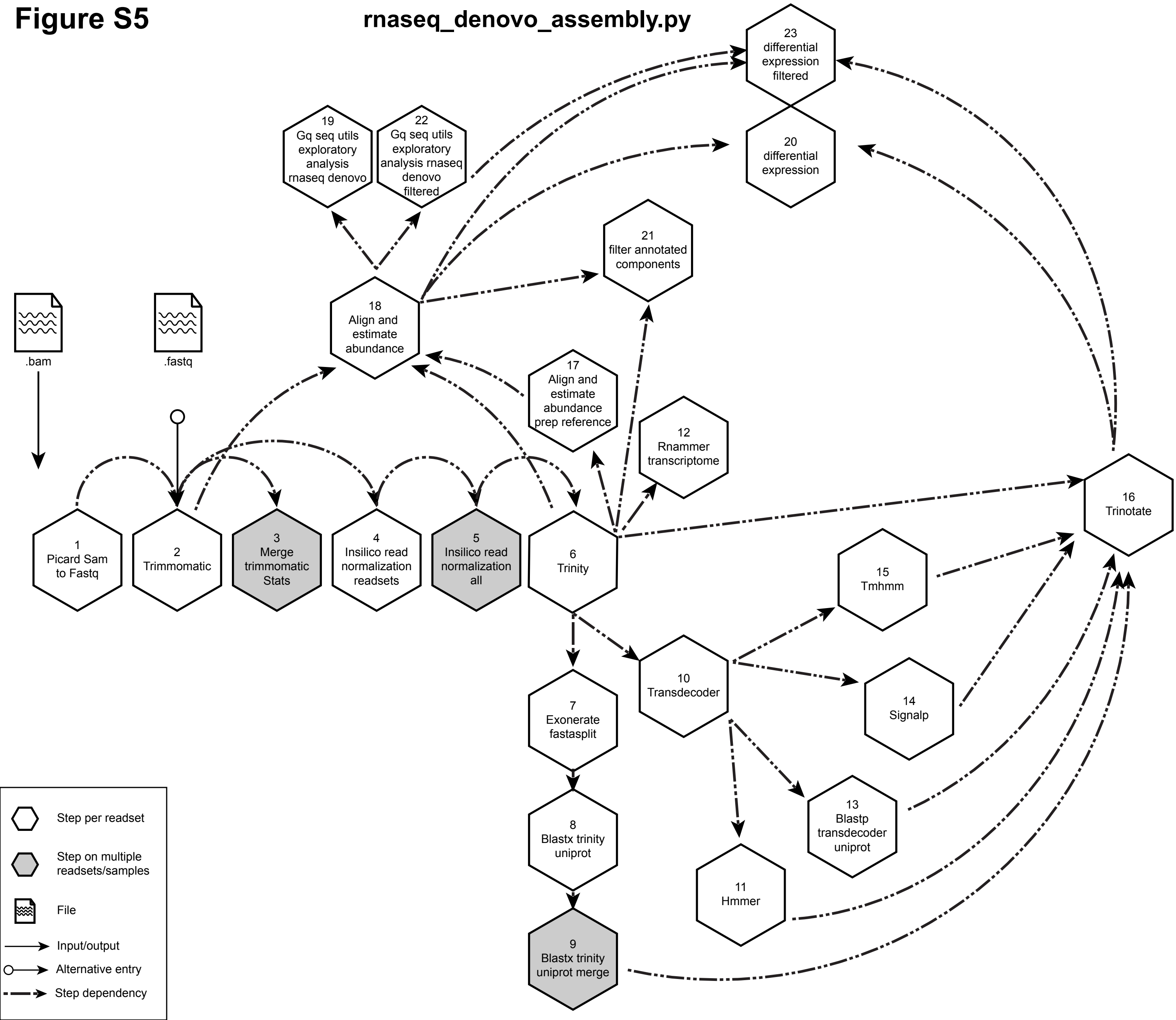
rnaseq_denovo_assembly.py
.bam
.fastq
1 Picard Sam to Fastq
2 Trimmomatic
3 Merge trimmomatic Stats
4 Insilico read normalization readsets
5 Insilico read normalization all
6 Trinity
7 Exonerate fastasplit
8 Blastx trinity uniprot
9 Blastx trinity uniprot merge
10 Transdecoder
11 Hmmer
12 Rnammer transcriptome
13 Blastp transdecoder uniprot
14 Signalp
15 Tmhmm
16 Trinotate
17 Align and estimate abundance prep reference
18 Align and estimate abundance
19 Gq seq utils exploratory analysis rnaseq denovo
20 differential expression
21 filter annotated components
22 Gq seq utils exploratory analysis rnaseq denovo filtered
23 differential expression filtered
Step per readset
Step on multiple readsets/samples
File
Input/output
Alternative entry
Step dependency

Figure S6

chipseq.py

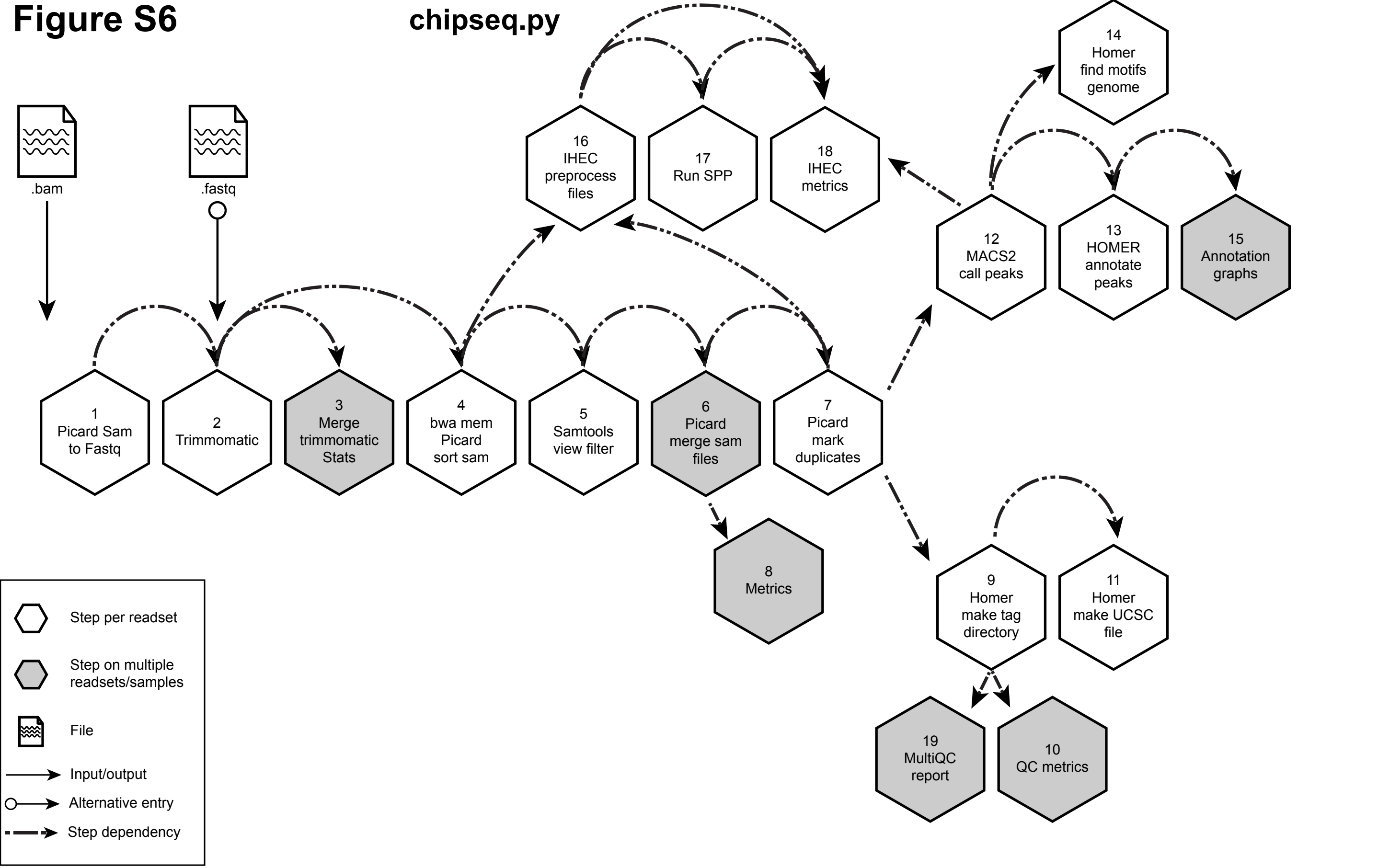

Figure S7

tumor_pair.py -t sv

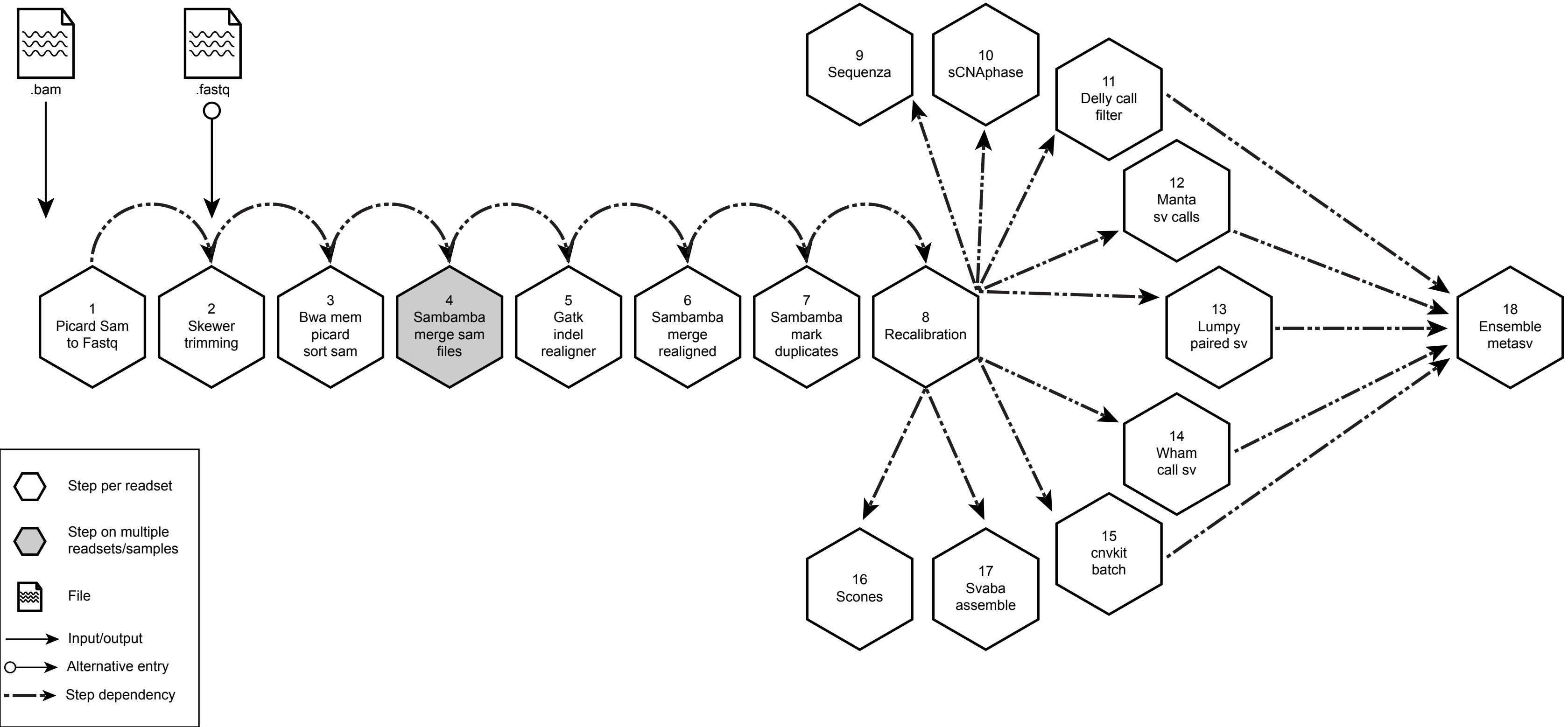

Figure S8

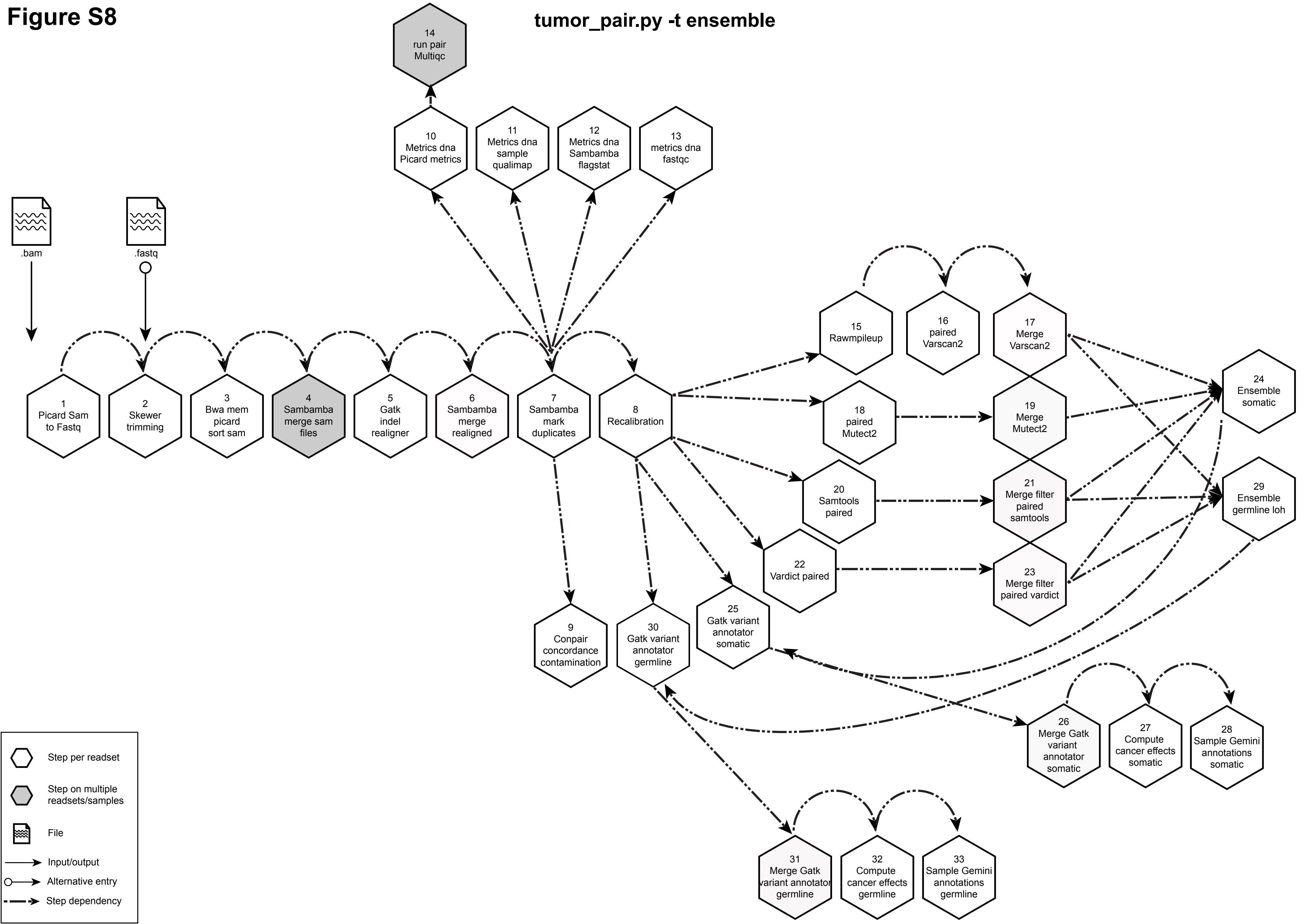
tumor_pair.py -t ensemble
.bam
.fastq
1 Picard Sam to Fastq
2 Skewer trimming
3 Bwa mem picard sort sam
4 Sambamba merge sam files
5 Gatk indel realigner
6 Sambamba merge realigned
7 Sambamba mark duplicates
8 Recalibration
9 Conpair concordance contamination
10 Metrics dna Picard metrics
11 Metrics dna sample qualimap
12 Metrics dna Sambamba flagstat
13 metrics dna fastqc
14 run pair Multiqc
15 Rawmpileup
16 paired Varscan2
17 Merge Varscan2
18 paired Mutect2
19 Merge Mutect2
20 Samtools paired
21 Merge filter paired samtools
22 Vardict paired
23 Merge filter paired vardict
24 Ensemble somatic
25 Gatk variant annotator somatic
26 Merge Gatk variant annotator somatic
27 Compute cancer effects somatic
28 Sample Gemini annotations somatic
29 Ensemble germline loh
30 Gatk variant annotator germline
31 Merge Gatk variant annotator germline
32 Compute cancer effects germline
33 Sample Gemini annotations germline
Step per readset
Step on multiple readsets/samples
File
Input/output
Alternative entry
Step dependency

Figure S9

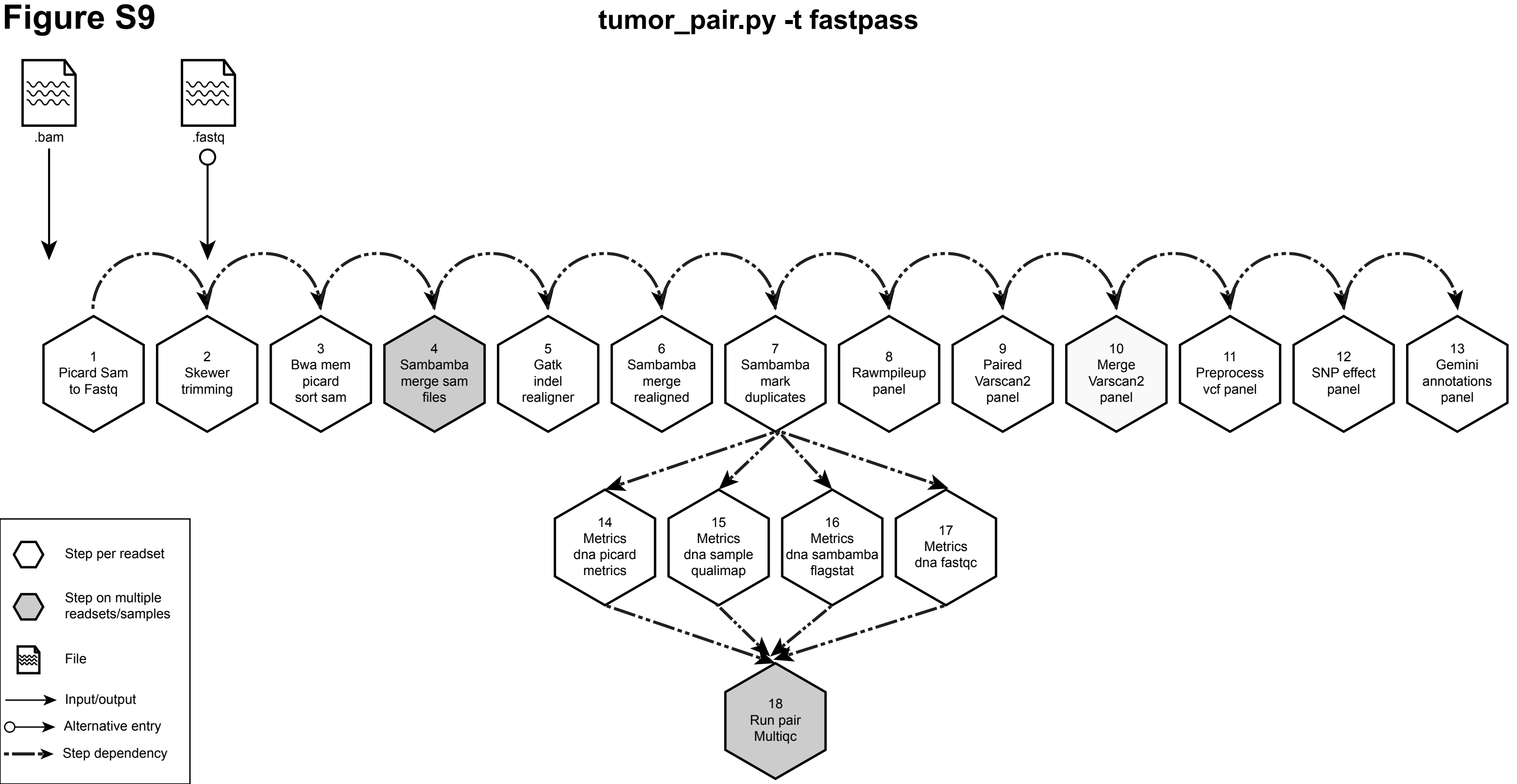
tumor_pair.py -t fastpass
.bam
.fastq
1 Picard Sam to Fastq
2 Skewer trimming
3 Bwa mem picard sort sam
4 Sambamba merge sam files
5 Gatk indel realigner
6 Sambamba merge realigned
7 Sambamba mark duplicates
8 Rawmpileup panel
9 Paired Varscan2 panel
10 Merge Varscan2 panel
11 Preprocess vcf panel
12 SNP effect panel
13 Gemini annotations panel
14 Metrics dna picard metrics
15 Metrics dna sample qualimap
16 Metrics dna sambamba flagstat
17 Metrics dna fastqc
18 Run pair Multiqc
Step per readset
Step on multiple readsets/samples
File
Input/output
Alternative entry
Step dependency

Figure S10

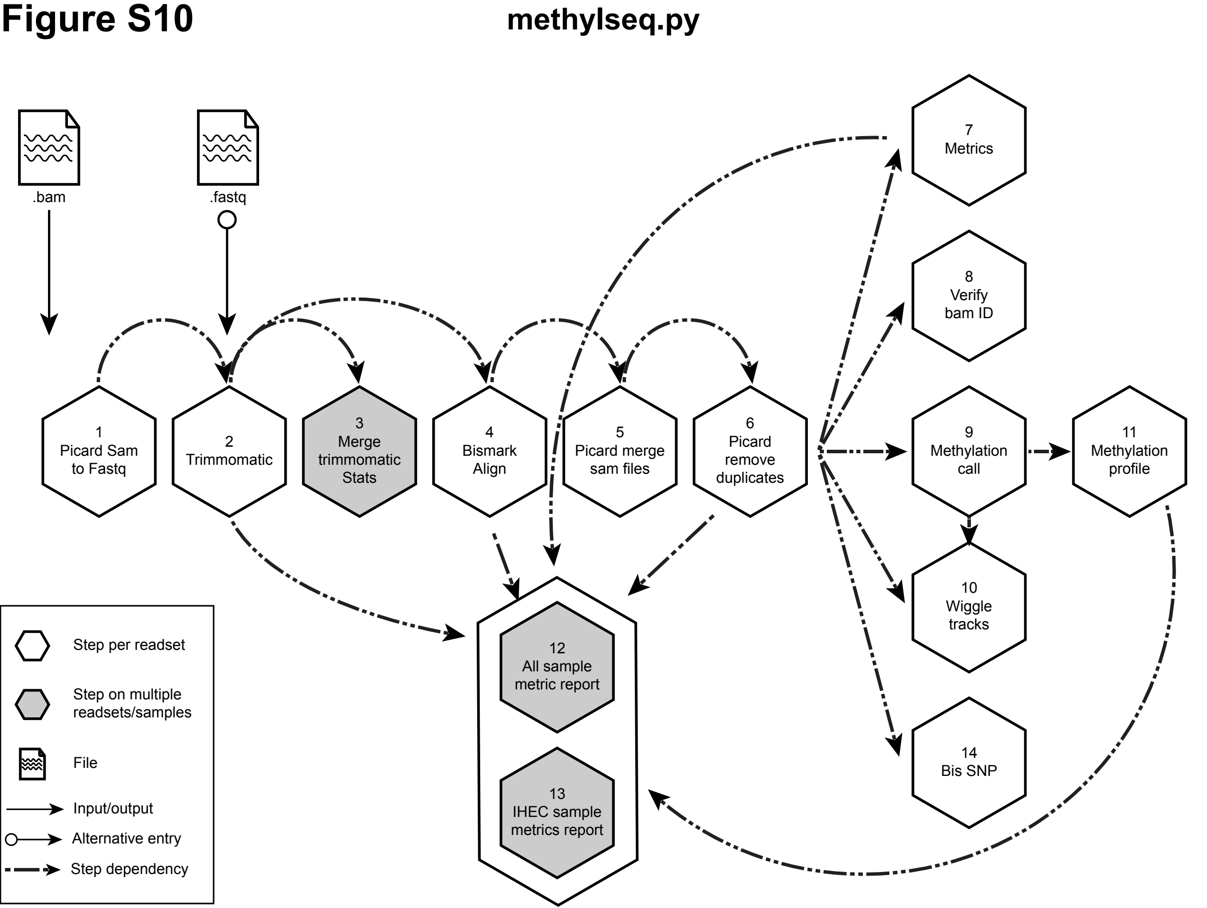
methylseq.py
.bam
.fastq
1
Picard Sam
to Fastq
2
Trimmomatic
3
Merge
trimmomatic
Stats
4
Bismark
Align
5
Picard merge
sam files
6
Picard
remove
duplicates
7
Metrics
8
Verify
bam ID
9
Methylation
call
10
Wiggle
tracks
11
Methylation
profile
12
All sample
metric report
13
IHEC sample
metrics report
14
Bis SNP
Step per readset
Step on multiple
readsets/samples
File
Input/output
Alternative entry
Step dependency

Figure S11

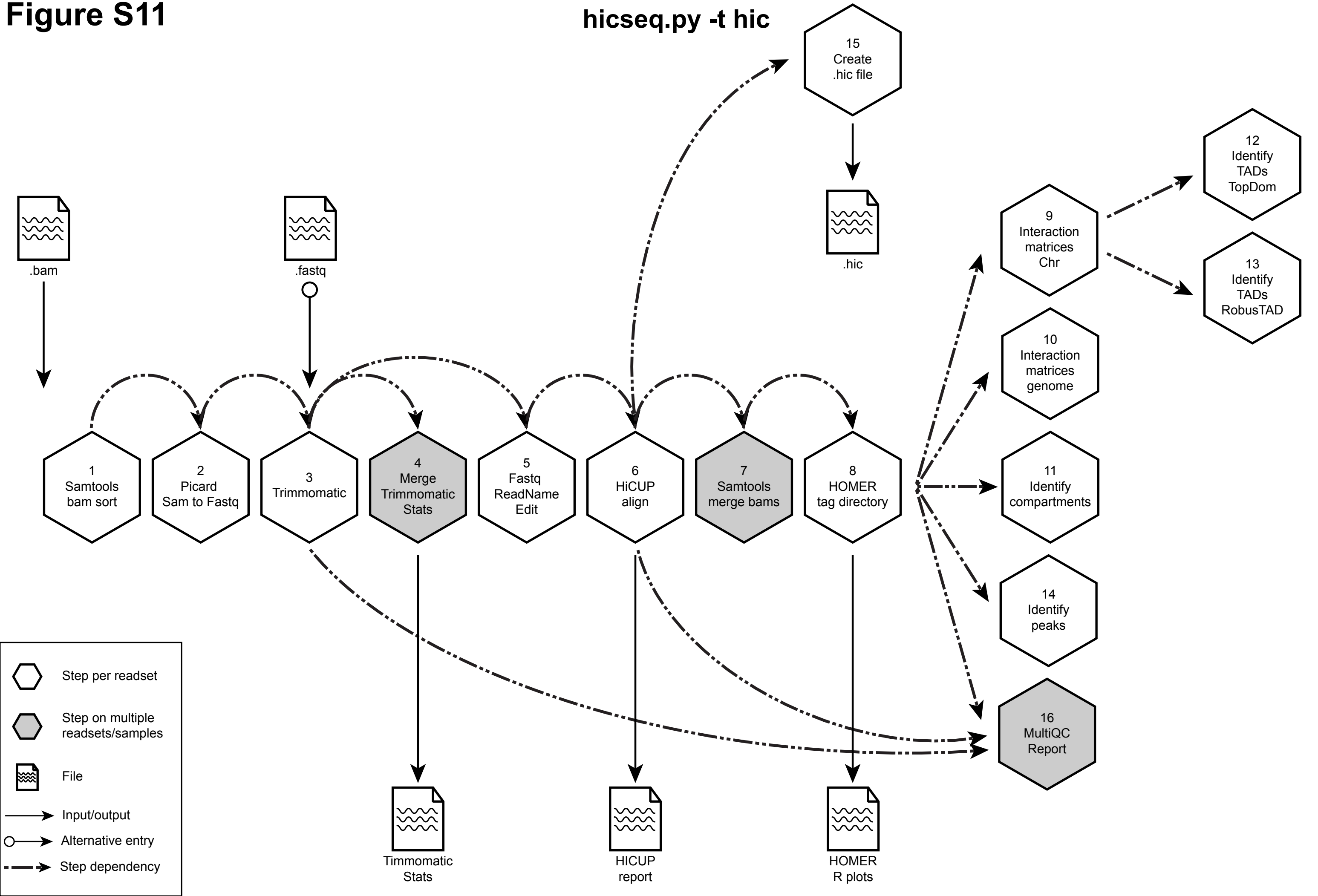
hicseq.py -t hic
.bam
.fastq
1
Samtools
bam sort
2
Picard
Sam to Fastq
3
Trimmomatic
4
Merge
Trimmomatic
Stats
5
Fastq
ReadName
Edit
6
HiCUP
align
7
Samtools
merge bams
8
HOMER
tag directory
15
Create
.hic file
.hic
9
Interaction
matrices
Chr
10
Interaction
matrices
genome
11
Identify
compartments
14
Identify
peaks
16
MultiQC
Report
12
Identify
TADs
TopDom
13
Identify
TADs
RobusTAD
Timmomatic
Stats
HICUP
report
HOMER
R plots
Step per readset
Step on multiple
readsets/samples
File
Input/output
Alternative entry
Step dependency

Figure S12

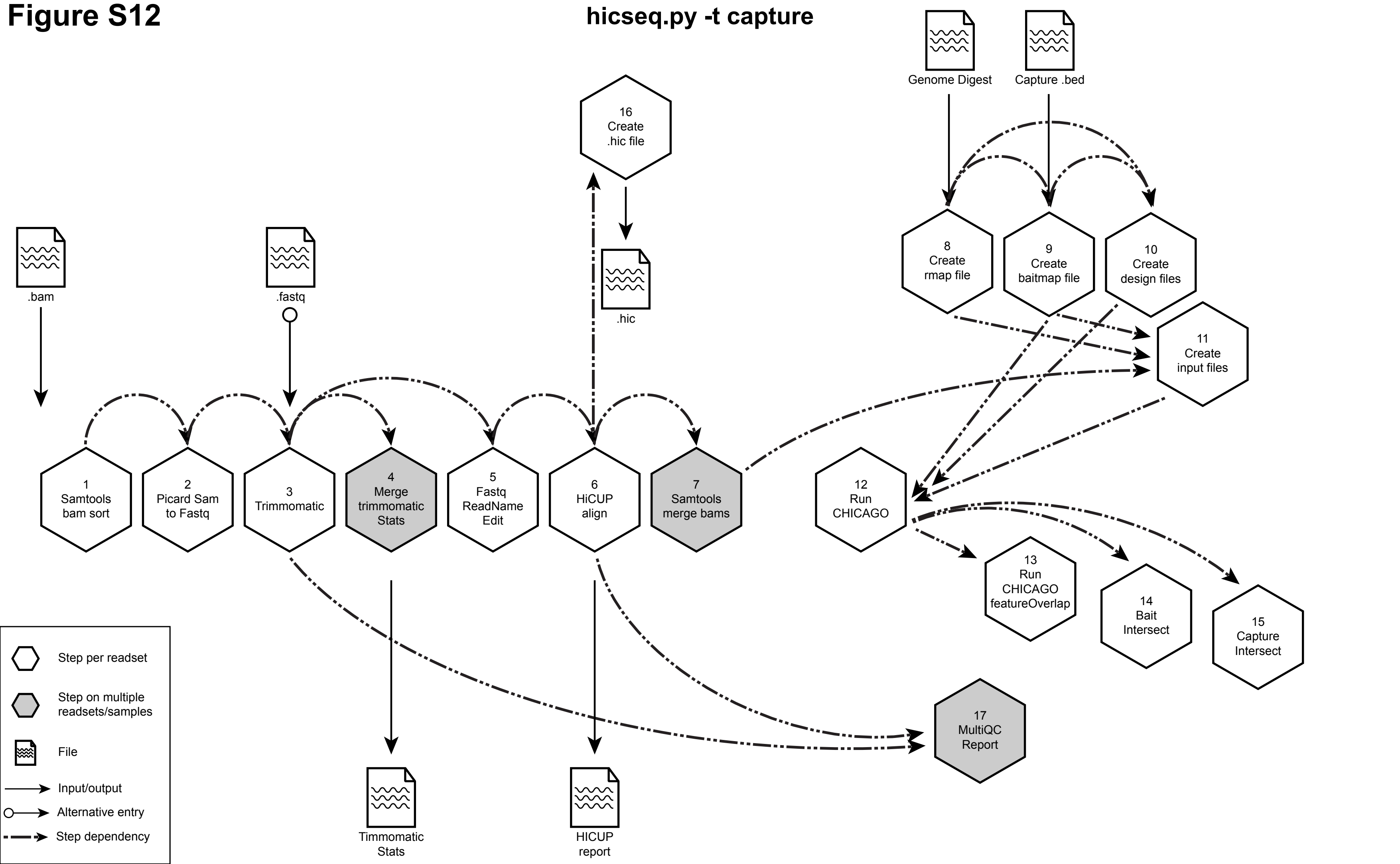

hicseq.py -t capture
Genome Digest
Capture .bed
16 Create .hic file
.hic
.bam
.fastq
8 Create rmap file
9 Create baitmap file
10 Create design files
11 Create input files
1 Samtools bam sort
2 Picard Sam to Fastq
3 Trimmomatic
4 Merge trimmomatic Stats
5 Fastq ReadName Edit
6 HiCUP align
7 Samtools merge bams
12 Run CHICAGO
13 Run CHICAGO featureOverlap
14 Bait Intersect
15 Capture Intersect
17 MultiQC Report
Timmomatic Stats
HICUP report
Step per readset
Step on multiple readsets/samples
File
Input/output
Alternative entry
Step dependency

Figure S13

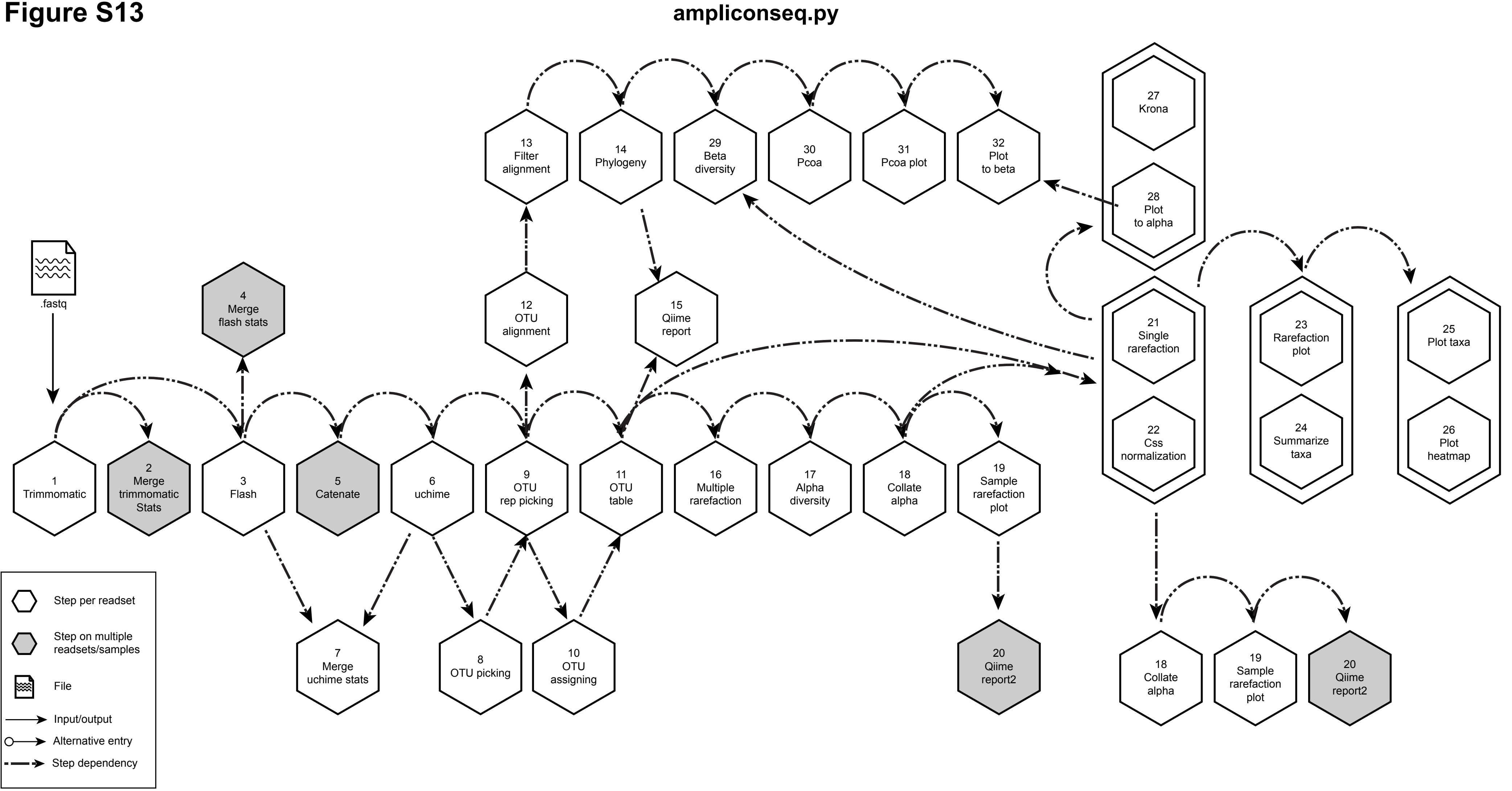
ampliconseq.py
.fastq
1 Trimmomatic
2 Merge trimmomatic Stats
3 Flash
4 Merge flash stats
5 Catenate
6 uchime
7 Merge uchime stats
8 OTU picking
9 OTU rep picking
10 OTU assigning
11 OTU table
12 OTU alignment
13 Filter alignment
14 Phylogeny
15 Qiime report
16 Multiple rarefaction
17 Alpha diversity
18 Collate alpha
19 Sample rarefaction plot
20 Qiime report2
21 Single rarefaction
22 Css normalization
23 Rarefaction plot
24 Summarize taxa
25 Plot taxa
26 Plot heatmap
27 Krona
28 Plot to alpha
29 Beta diversity
30 Pcoa
31 Pcoa plot
32 Plot to beta
18 Collate alpha
19 Sample rarefaction plot
20 Qiime report2
Step per readset
Step on multiple readsets/samples
File
Input/output
Alternative entry
Step dependency

Figure S14

pacbio_assembly.py

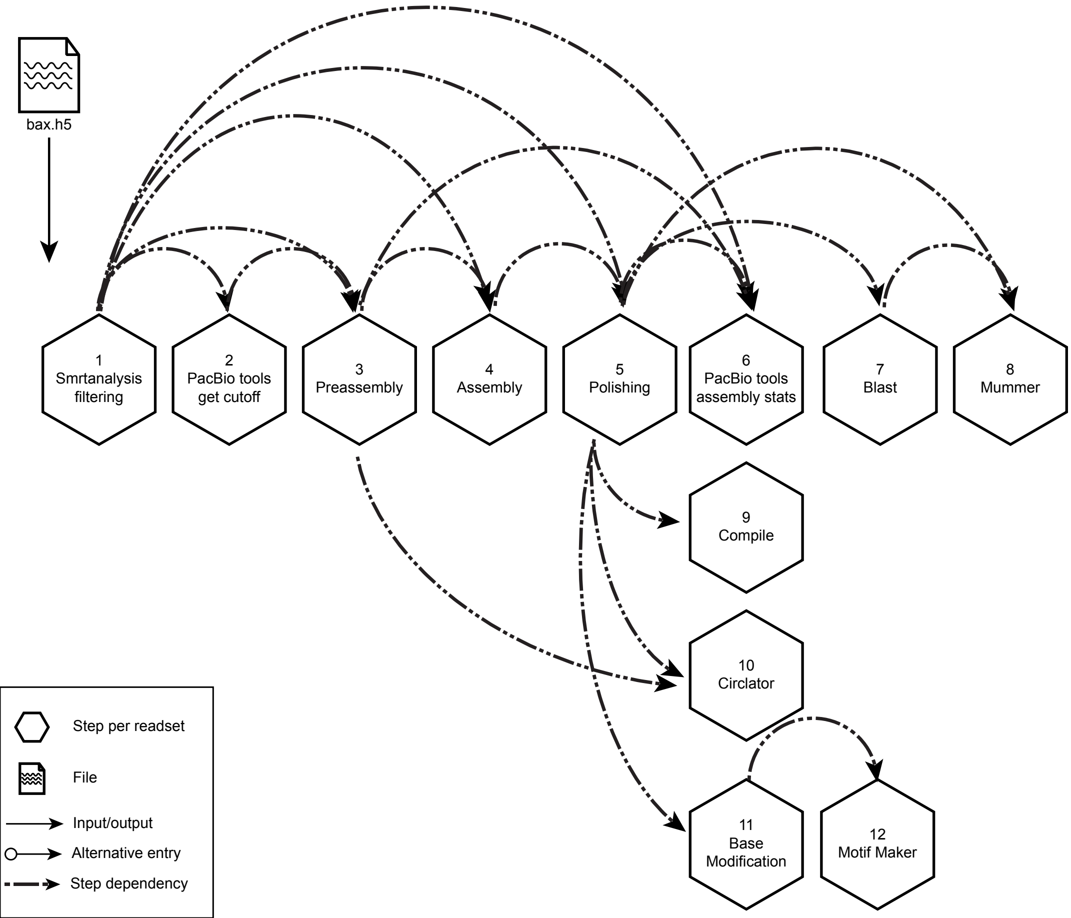

Supplement: giz037_Supplemental_File [file giz037_supplemental_file.pdf]
