# Supplementary material for: GenPipes: an open-source framework for distributed and scalable genomic analyses
Source: Gigascience. 2019 Jun 11;8(6):giz037. doi: 10.1093/gigascience/giz037 (PMC6559338; doi:10.1093/gigascience/giz037)
Supplement: giz037_Response_to_Reviewer_Comments_Original_Submission [file giz037_response_to_reviewer_comments_original_submission.pdf]

Author's Response To Reviewer Comments

Close

Dear GigaScience Editorial Office,

Thank you for opportunity to submit a revised version of the manuscript GIGA-D-18-00198 that addresses the points raise by the two reviewers. Following the constructive comments and suggestions, we have made several major improvements the manuscript, including:
- The reviewers had comments about the dependencies between steps, as well as whether steps worked on single sample or a cohort of samples. To help answer these questions, we have added a diagram summarizing each pipeline to the supplementary.
- We have added extensive benchmark metrics for two pipelines, dnaseq and tumour_pair.
- We have added documentation on how to run GenPipes on a cloud platform on our website and now referenced it in the manuscript.
- GenPipes is now registered in the SciCrunch database under the RRID SCR_016376. The RRID has been included in the revised manuscript under the 'Availability and requirements' section.
- We have re-organized the text a bit to better explain the unique features of GenPipes

See below for our point-by-point response to the reviewers. New text that has been added is shown in red in the revised manuscript.

Response to the Reviewers:
Reviewer #1: The authors present in this manuscript both a new workflow management system (GenPipe), as well as a set of bioinformatics pipelines that are built to run on this system. The authors contribution is likely be of interest to man genome centres and bioinformaticians, who wish to leverage existing pre-built and tested pipelines. The manuscript is clear and well written and the source code is well structured, extensive, and is well documented. The developers have also taken steps to ease installation and configuration issues that might occur when trying to install the software in other environments.
However, I have reservations regarding the structure and content of the manuscript. I find it lacks detail and analysis that would convince a reader to adopt their system, both with regards to GenPipe itself, as well as the pipelines. This is unfortunate as I think the authors have provided a large contribution to the field in making available their resources.
We thank the reviewer for evaluating the manuscript and for his positive view of our contribution to the bioinformatics community. We have now re-organized the text a bit to better explain the unique features of GenPipes and have added more information to the manuscript that should provide the users with a better overview of its advantages (see below).
Major points:
1. The authors only provide a superficial comparison to existing systems. A more detailed analysis of why new pipeline developers should use GenPipe over an alternatives? What distinguishes this as a WMS from SnakeMake for example? From what I can see in the manuscript there are several implementation details within GenPipe that appear sub optimal, which I'll elaborate on in the points below.

In our original submission, we attempted to provide a comparison of GenPipes to other available WMSs. While far from exhaustive, we had compared GenPipes to 18 popular WMSs by looking at 9 different features and 9 different pipelines (Table 1). In the revised submission, we have added SnakeMake to the WMS list and included 3 more features suggested by reviewer #2 (see below). We have also added a detailed workflow for each pipeline (Figure 3 and S1-14) and have added new benchmarking metrics for some of the pipelines. We also included estimate resource usage for each pipeline across different servers (Table S1). We have also moved the description of the unique features of GenPipes to the Result section of the manuscript. We think that this new information will hopefully give potential users a better idea of how GenPipes compares to some of the existing frameworks available and its advantages.
2. I would also like to see a proper analysis for each pipeline (can be provided in supplemental information) describing comparisons to existing pipelines in terms of accuracy, resource usage, runtime stats, etc.

We agree with the reviewer that benchmarking the individual pipelines for accuracy, resource usage and runtime statistics is important. Along those lines, we have added benchmarks for several pipelines to the Supplementary Material. In terms

resource usage and runtime statistics, it has been our experience that these metrics vary widely depending on system hardware, software versions and sample sequencing depth. However, we agree that a ballpark estimate of these resource would be useful for the user and have added Table S1.

In terms of accuracy, it is important to remember that GenPipes is a framework that is built around open source, third party tools that are available to the scientific community. Accuracy is not as easily assessed in certain fields due to the lac of a good quality "truth" set to benchmark against. Generally, we have tried to model GenPipes pipelines following large scale projects like GATK best practices SOPs and ENCODE and have relied on public benchmarking, in addition to our own

Minor points:

1. Introduction: "Such solutions are flexible and can help in pipeline implementation but do not provide robust standardized pipelines which are ready for production-scale analysis." In my experience, it's simply not true that WMS solutions are not suitable for production scale analysis. There are many examples of people doing exactly this, and moreover I've built several myself which are run multiple times every day without issue. In my experience they can work very reliably, and ability to tolerate and resume from errors is easy to code in. It is also unclear what the authors mean b standardisation in this context. I'd request that the authors either justify this point and provide concrete examples of exactly what the source of the perceived issues are, or remove this sentence.

We agree with the reviewer that the sentence is not delivering the idea we intended. The sentence has been edited in the manuscript to highlight the fact that not all WMSs come with pre-built pipelines that are ready for use. We agree with the reviewer that many WMSs are robust and powerful. We are simply trying to appeal to both the advanced user with GenPipes's WMS and the novice user with the pre-built and tested pipelines.

The sentence now reads:
"Such solutions are flexible and can help in pipeline implementation but rarely provide robust pre-built pipelines which are ready for production analysis."

2. Introduction: "These are useful for specific applications but can be challenging to implement, difficult to modify or scale up. They have also rarely been tested on multiple computing infrastructures." This seems too strong a statement. In son cases this might be true but there are many examples of robust pipelines that efficiently leverage data centre hardware.

We agree with the reviewer that the statement might be generalized beyond context. We have edited the text to explicitly prevent the statement from applying to all pipelines.

The sentence now reads:
"These are useful for specific applications but can sometimes be challenging to implement, difficult to modify or scale-up."

3. Introduction: "GenPipes has been tested, benchmarked ...". It is not clear whether the "testing and benchmarking" refers to the pipelines or the WMS itself. This should be clarified.

Both GenPipes's pipelines and the WMS have been tested extensively. The pipelines have been benchmarked and have been used to process thousands of samples. The WMS has been stress-tested and adapted to different computing infrastructure and is currently run on at least 6 super computers that we help maintain. We have modified the text to clar this.

The sentence now reads:
"GenPipes' WMS and pipelines have been tested, benchmarked and used extensively over the past four years."

4. Schedulers: Ideally, GenPipes should offer the ability to implement scheduling via DRMMA which would increase the potential sites that could potentially run genpipe. For example, currently any data centres running Platform LSF could not use genpipe but via4 DRMMA this would be possible.

We thank the reviewer for his suggestion. We generally like to minimize the number of layers between GenPipes and the system it is running on, and did not consider DRMAA previously. We have added the DRMAA scheduler to our potential future projects and have mentioned it in the discussion.

5. Job dependencies: I have reservations that the approach taken here is optimal. If I understand correctly, job dependencies are setup using the selected scheduler and all jobs, across steps, are launched at the same time. I suspect for very large pipelines containing many thousands jobs (not uncommon) this would put an undue burden on the schedule and therefore would not scale very well. Could the authors elaborate on this point and highlight details such as what happens when a pipeline fails? Are existing jobs explicitly terminated? Or somehow left running and continue after the pipeline is resumed?

We agree with the reviewer that this feature may be optimized and have been working on this for a future GenPipes release. It is not ready yet, as we would like to optimize and test all supported schedulers before releasing the new featur The current process works by creating the full script which is communicated to the scheduler. This approach has been

initially chosen for its low level of complexity and reproducibility; the script is readable and editable by a minimally trained user and can also be re-run later on. This also avoid having to generate local processes which would need to stay in active mode in order to monitor the job submission process. In order to help monitoring the pipeline progress, GenPipes includes a script (logReport.pl) that generates a report of the current status of the pipeline. We have also included a JSON log file system that could be used to develop a local web-portal displaying the pipeline job status in real time.
We have been using GenPipes on hundreds of samples every day, submitting thousands of jobs, and has not run into any issues with our compute providers so far. However, we do intend to optimize the process by using job arrays.

In terms of what happens when the pipeline fails, we have added a section to the text under "Running GenPipes" to elaborate on this point:

"… Once launched, the jobs are sent to the scheduler and queued. As jobs complete successfully, their dependent jobs are released by the scheduler to run. If a job fails, all its dependent jobs are terminated and an email notification is sent to the user. When GenPipes is re-run, it will detect which steps have successfully completed, as described in section 'Smart relaunch features', and skip them but will create the command script for the jobs that were not completed successfully..."

6. Configuration Files: "Configuration files, also referred to as "ini" files, are provided among the arguments of the GenPipes command.". The authors should change the wording here. "Ini" is a legacy windows-based configuration file format. I'm not asking for the authors to change the configuration format used but it would be useful to have some justification for this unusual choice. Alternatives, like "yaml" for example allow for stricter and richer structuring and is therefore much easier to parse and in turn normally results in less buggy code.

We agree with the reviewer that many file configuration formats exist and that some might be considered more optimal than the legacy ini file format. However, we chose the ini format for its readability and the ease of use of the ini schema (section, key and value). It can be edited manually with any text editor without the need to worry about syntax or indentation which is easier for standard analysts using GenPipes for their analysis. Additionally, the python language offers a standard library (configParser) that is made to easily integrate this standard configuration format.

Reviewer #2: The manscript presents GenPipes, a Python-based framework for defining and executing data analysis workflows.

GenPipes is based on a handful of Python classes that can be inherited and implemented to achieve a formal and executable description of a workflow in terms of steps. During execution, steps are specialized to jobs that perform concrete operations on input files.

For me, the most important, and definitely valuable addition of this work is the comprehensive collection of well-tested workflows covering the most important applications of sequencing.

In general, I think this should be emphasized more, at the expense of removing some of the weaker aspects of the paper. I will outline this below.

Major Comments

* The manuscript argues that a major advantage of GenPipes is the rich collection of production-ready workflows that are delivered with the system. The list of workflows is indeed impressive, it should be mentioned though that both Snakemake and Nextflow also provide (community-maintained) collections of tested workflows, like github.com/snakemake-workflows, nf-core.github.io and sequana. I agree though that it might very well be that these are still less mature (except sequana), as they are probably newer.

We thank the reviewer for seeing value in our growing collection of pipelines. In our manuscript, we avoided reference to community-maintained workflows. While community-maintained workflows are a great testament to the usefulness of a WMS, they are hard to keep track of and evaluate. GenPipes supports community-maintained workflows as well, however, those too have not been mentioned in the manuscript. The pipelines that have been mentioned are pipelines that have been validated and are maintained by the tool authors, which we think is an important distinction.

We have made this clear in the text, as follows:
"It is important to note that GenPipes, as well as several other WMSs, have community-supported pipelines, however, those have not been included in the comparison."

* The manuscript claims that GenPipes supports cloud execution, but I cannot find a scheduler for this purpose in the list of schedulers on page 4. Also, the feature table says that cloud support is pending.

Yes, GenPipes supports cloud execution via a container image and not a particular scheduler. Through the container image any available scheduler can be used, depending on the cloud architecture in place. We apologize for the omission in Table we have fixed it. We have now also documented the use of GenPipes in the cloud and added a user manual at:

http://www.computationalgenomics.ca/genpipes-in-the-cloud/
* On page 7, when describing deployment of software and reference information, it is unclear whether installation happe system wide (needing admin rights) or local. This should be clearly stated, since system-wide installation would be a majo disadvantage compared to systems like Nextflow, Snakemake or CWL based WMSs. Moreover, it should be mentioned how those dependencies are updated, and in what sense such updates would affect previous runs, which could potentially loose reproducibility, if updates happen globally.

GenPipes installation can happen both system-wide or locally as the pipeline will only use software and modules provided a specific path defined through an environment variable. All the third part tool installation scripts provided with the pipelir have been designed to work on a local path system and do not require root privileges. We also developed a container imag of GenPipes which runs GenPipes with little software installation. This allows larger processing centers to install GenPipes for all users, but also allows individuals the flexibility to adopt GenPipes for their own needs without needing special permissions or setup.

We have added text in the result section to explain these points and to expand on the dependency updates:

“These scripts support local installations without the need for super-user privileges. Tools and dependencies are versioned and are loaded by GenPipes in a version-specific manner. This allows different pipelines to use different software versions based on need. It also allows retention of the same parameters and tools for any given project for reproducibility. GenPipe is also provided as a container version for which no dependency installation is required.”
* In the discussion, it is mentioned that GenPipes is currently being reimplemented in WDL. It is a good choice to use one of the established, more feature-rich systems. However, then, large parts of this paper are in fact obsolete, as they will be replaced with WDL. The major contribution that remains after that step is the collection of workflows, which is totally fine, since this is a very valuable addition. I therefore suggest to put more focus on the workflows, and simply outline that they are currently implemented in GenPipes and soon will be avaialble in WDL. Morevoer, choice of tools, parameters and how the benchmarking was done (in a more concrete way instead of simply saying "we used GIAB") should be described in detail.

Actually, based on recent developments, we are probably going to go with CWL over WDL. That being said, we do not fully agree with the reviewer that this will make GenPipes WMS obsolete. While CWL or WDL can help increase the compatibility of GenPipes with different systems, it will add a layer of complexity to GenPipes; one that is not needed on HPC systems. We agree with the reviewer that the pipelines are a major contribution of GenPipes and that they deserve a more detailed description. We have now added more descriptions and benchmarks as supplementary material. We have also added workflow diagrams representing pipeline workflow and dependencies in the supplementary.

* Table 1 provides a feature comparison. As with every single feature comparison I have seen so far, it is highly biased, showing only features that GenPipes itself provides. For example, GUI (as provided e.g. by Galaxy) and automatic reports are missing. Per-step/job software deployment and container support is missing. Config file validation is missing. Items ar not sufficiently explained (e.g., what is meant with tracking, and in what sense is Nextflow not providing it). A popular system is completely missing from the table: Snakemake. Via nf-core and other projects, Nextflow and Snakemake provic several of the mentioned pipelines. Finally, I cannot actually find that table in reference [62], although the authors claim that it is a modified version of the table from that paper.

We thank the reviewer for his excellent suggestions. We have added 3 more features to the comparison table (GUI, Repor and Config validation). We have also added SnakeMake to the comparison. In evaluating the pipelines available, we have looked at the published manuscript if it exists or the code base and documentation of each tools. Community-maintained pipelines were not considered, as it becomes difficult to draw the line on what to include in the comparison and it is hard t assess the reliability of these pipelines without extensive benchmarking. Only pipelines provided by the tool authors were considered.
Finally, the comparison Table1 was modified from Griffith et al. in supplementary material (Table S6) which contains a simple version of the table. following the link in the figure leads to the full version of the table:
https://journals.plos.org/ploscompbiol/article/file?id=info%3Adoi/10.1371/journal.pcbi.1004274.s021&type=supplementa
Minor Comments
* On page 2, when mentioning other WMSs, the authors should also mention Nextflow. Moreover, CWL and WDL are no WMSs, and should be listed separately as "declarative workflow description languages".

We have edited the text as suggested.
* On page 5, when relaunch features are mentioned, common functions of other systems like manual forcing or handling of missing files are not mentioned. Are these not available?

GenPipes supports manual forcing through the “-f” option. GenPipes validates the existence of all required modules and

genome files and input files in the config file before creating the commands. If any are missing, it looks for alternative file[illegible] using the ordered list of input implementation described in the “Key GenPipes features options”. If none of the possible fil[illegible] are found, GenPipes will throw a Missing File exception and terminate. The text has been edited to clarify this.

* On page 6, the description of input choice does not really make it clear how multiple input files or aggregation is handled. It would be beneficial to see examples for (a) a 1-in-1-out job, (b) an aggregating job, (c) a scattering job, (d) a [illegible] mixed job (n-in-m-out).

GenPipes has an array of steps with different behaviors. Some steps operate on a single sample input while others operat[illegible] on the cohort of available samples (metric steps). To try to distinguish these, we added color coding (back/white) to the workflow diagrams we added in supplementary Figure 1. The dependencies between steps are mapped by GenPipes through input and output files required and communicated to the scheduler which then coordinated job launch. For a full li[illegible] of pipelines and steps, please refer to Figure S1-14.

* On page 8: "all workflows acceps a bam or fastq file as input". I guess they accept multiple bams or fastqs, right? Otherwise they could only be applied to a single sample at a time...

To take full advantage of HPC power and reduce processing times, GenPipes runs each sample separately, when possible. Some steps aggregate inputs from many samples at a time. Those have been colored in black in supplementary Figure2 S1-14.

Close
