# Supplementary material for: GenPipes: an open-source framework for distributed and scalable genomic analyses
Source: Gigascience. 2019 Jun 11;8(6):giz037. doi: 10.1093/gigascience/giz037 (PMC6559338; doi:10.1093/gigascience/giz037)
Supplement: giz037_Response_to_Reviewer_Comments_Revision_1 [file giz037_response_to_reviewer_comments_revision_1.pdf]

Author's Response To Reviewer Comments

Close

Dear Editor,

Thank you for the opportunity to submit a revised version of the manuscript GIGA-D-18-00198, which addresses the final points raised by the reviewers. Please find our point-by-point response below. New text that has been added to the revised manuscript is shown in red.

Response to the Reviewers:

Reviewer #1: I thank the authors for taking the time to address my previous comments. I believe the manuscript is much stronger as a result and I have no further comments to add.

We thank the reviewer for his constructive criticism that has strengthened the manuscript.

Reviewer #2: The authors successfully address various of my and my colleagues requests. However, certain issues remain, which I will list in the following:

Major

* In the introduction, the authors say that frameworks like Galaxy can be inconvenient on large scale projects. Why is that? I think such a claim should be support by a detailed reasoning.

Frameworks like Galaxy are generally web-based. For large scale projects, uploading large datasets to a platform can be time/resource intensive. In general, when projects get larger, it is more efficient to bring the software to the data and not upload the data to the software location.

We have adjusted the text to say:

"… such tools can be inconvenient for large scale projects due to having to move sizeable datasets to the platform".

* When mentioning that WMSs rarely provide pre-built pipelines ready for production analysis, the authors should also mention that they nevertheless support development of such pipelines by the community of users, including linking out to examples like nf-core and github.com/snakemake-workflows.

We have edited the text accordingly:

"It is important to note that GenPipes, as well as several other WMSs, like Nextflow [58] and SnakeMake [59], support community-developed pipelines, however, those have not been included in the comparison."

* In my previous comment, I mentioned that the feature table is biased. While the authors added the columns suggested, these where only meant as examples. I would have thought that the authors take this as incentive to get a less biased view, which is arguably very hard. However, even when only taking the reviewer comments as a base, there are plenty of other columns which should go into the table. For example, the authors should add "DRMAA support", "status/progress monitoring" as a column. Moreover, the level of cloud support in GenPipes is quite different from what is offered by e.g. Nextflow and Snakemake. There, you have full Kubernetes support, in case of Snakemake even without the requirement of a shared filesystem. Maybe split the cloud column into "basic cloud support" and "kubernetes support".

Our intentions with Table1 was to provide the reader with an overview of the features of several tools in the field but not necessarily an exhaustive list. We did not design the table to be biased towards GenPipes as we only modified one of the most comprehensive tables we found in recent manuscripts (Griffith & Griffith et al.). Based on that initial table and reviewers' comments, we added 3 features and 1 WMS. Although more could be added, it would also start cluttering the table and make it difficult to extract meaningful information.

"status/progress monitoring" is already included in the table under "Tracking". We have modified the column name to make it less ambiguous.

Concerning "DRMAA support" and splitting the "cloud support" column into "basic cloud" and "kubernetes support", we feel that this is highly technical/specific for the average user.

* The installation mechanism for new software tools (outside of what is provided out of the box) (explained here: https://bitbucket.org/mugqic/genpipes/src/master/#markdown-header-modules), seems like manually redoing all the work that is already solved by package managers like conda or container engines like singularity. For example, Bioconda provides a library of over 4000 bioinformatics software packages which can be readily used from any WMS that supports conda, and Biocontainers provides the same for container based deployment (which lacks conda's ability to rapidly compose custom combinations of tools though). In order to make the comparison fair, the feature table should therefore contain two columns called "package-manager-integration" and "container-integration". For an example of what level of integration I am referring to, see https://snakemake.readthedocs.io/en/stable/snakefiles/deployment.html#integrated-package-management and https://www.nextflow.io/docs/latest/conda.html?highlight=conda.

Bioconda offers a collection of packages and not an integrated system and can be quite heavy in memory requirements. Hence, we think that "package-manager-integration" is not necessarily an indication of the strength of the WMS. It is a specific choice, one that offers ease of installation but has its pitfalls as well. GenPipes does not use package managers by design. GenPipes manages its own libraries making sure there is no conflicting libraries in the process. For users who do not want to install GenPipes manually, we offer a Docker container that has also been tested with Singularity. We have updated the GenPipes' bitbucket documentation to highlight the availability of the GenPipes' Docker container.

"container-integration" has already been included in the table under the "Cloud/Container" column.

* I am pleased to see that GenPipes indeed supports aggregation over many samples. What remains is the question whether the only entity to aggregate over are samples. If so, only over all samples or is it possible to express e.g. an arbitrary grouping of samples? Moreover, what about other properties, e.g. for scanning a parameter space? I suggest to somehow reflect the different ways of aggregation in the feature table, maybe using the terms that I mentioned in my first review.

GenPipes is a flexible python framework that aggregates over readsets, samples and other entities, like chromosomes, based on the pipeline. Arbitrary groupings of samples can be defined in pipelines that use design files, like chipseq and rnaseq. Scanning parameter space can be done by adjusting the configuration files. There isn't a set of limited/defined aggregation methods we use; aggregation is used based on each pipeline's needs. The user can refer to the documentation of each pipeline to see what is possible. For user implemented pipelines, there is no restriction on the aggregations possible.

We have added the following lines to the text to highlight some of these points:

"… GenPipes can aggregate and merge samples as indicated by the readset file."
"… Configuration files are customizable, allowing users to adjust different parameters."
"… Custom sample groupings can be defined in the design file."

Minor
* Please mention in the caption of the feature table that community based workflows are not considered in the comparison. It might otherwise be that readers overlook this in the main text.

We have added test to the caption as follows:

“Modified from Griffith & Griffith et al. [57]. Note that community-built pipelines are not considered in the Pipelines section of the table.”

* Figure S1 contains a lot of typos, e.g. "reasdet", which I guess is supposed to be readset?

Thank you, we have corrected the typos in Figure S1.

Close
