# Supplementary material for: GenPipes: an open-source framework for distributed and scalable genomic analyses
Source: Gigascience. 2019 Jun 11;8(6):giz037. doi: 10.1093/gigascience/giz037 (PMC6559338; doi:10.1093/gigascience/giz037)
Supplement: giz037_Response_to_Reviewer_Comments_Revision_2 [file giz037_response_to_reviewer_comments_revision_2.pdf]

Author's Response To Reviewer Comments

Close

Dear Editor,

Thank you for the opportunity to submit a revised version of the manuscript GIGA-D-18-00198, which addresses the final points raised by the reviewers. Please find our point-by-point response below. New text that has been added to the revised manuscript is shown in red.

Response to the Reviewers:

Reviewer #2: I thank the authors for addressing several of my concerns. Two issues remain:

Major comments

* Regarding the feature table, the reasoning of the authors is acceptable (apart from my concern below), if the caption explicitly mentions that the table is "meant to provide the reader with an overview of the features of several tools in the field but not necessarily an exhaustive list".

We have added the suggested text to Table 1's caption as follows:

"… It is also worth noting that the following table is meant to provide the reader with an overview of the features of several tools in the field but not necessarily an exhaustive list. For a full description of each tool's capabilities, please consult their official documentation."

* Regarding the answer "Bioconda offers a collection of packages and not an integrated system and can be quite heavy in memory requirements. Hence, we think that "package-manager-integration" is not necessarily an indication of the strength of the WMS. It is a specific choice, one that offers ease of installation but has its pitfalls as well. GenPipes does
not use package managers by design. GenPipes manages its own libraries making sure there is no conflicting libraries in the process. For users who do not want to install GenPipes manually, we offer a Docker container that has also been tested with Singularity. We have updated the GenPipes' bitbucket documentation to highlight the availability of the GenPipes' Docker container":

The way to provide the software stack for an analysis is very important for reproducibility and maintainability of a pipeline. Hence, this should definitely be a column of the feature table in any case. Certainly it is legitimate to decide against Conda or other package managers, but in above argument I do not find any reason to hide this aspect from the user in the feature table. Why not adding a column about package manager support and let the reader decide whether they need/want this feature or not?

We have added a column, "Package Manager" , to Table1, as suggested by the reviewer.

Close
