# Supplementary material for: GenPipes: an open-source framework for distributed and scalable genomic analyses
Source: Gigascience. 2019 Jun 11;8(6):giz037. doi: 10.1093/gigascience/giz037 (PMC6559338; doi:10.1093/gigascience/giz037)
Supplement: giz037_Reviewer_1_Report_Original_Submission — Daniel Mapleson -- 6/21/2018 Reviewed [file giz037_reviewer_1_report_original_submission.pdf]

Reviewer Report

Title: GenPipes: an open-source framework for distributed and scalable genomic analyses

Version: Original Submission Date: 6/21/2018

Reviewer name: Daniel Mapleson

Reviewer Comments to Author:

The authors present in this manuscript both a new workflow management system (GenPipe), as well as a set of bioinformatics pipelines that are built to run on this system. The authors contribution is likely be of interest to many genome centres and bioinformaticians, who wish to leverage existing pre-built and tested pipelines. The manuscript is clear and well written and the source code is well structured, extensive, and is well documented. The developers have also taken steps to ease installation and configuration issues that might occur when trying to install the software in other environments. However, I have reservations regarding the structure and content of the manuscript. I find it lacks detail and analysis that would convince a reader to adopt their system, both with regards to GenPipe itself, as well as the pipelines. This is unfortunate as I think the authors have provided a large contribution to the field in making available their resources.

Major points:

1. The authors only provide a superficial comparison to existing systems. A more detailed analysis of why new pipeline developers should use GenPipe over an alternatives? What distinguishes this as a WMS from SnakeMake for example? From what I can see in the manuscript there are several implementation details within GenPipe that appear sub-optimal, which I'll elaborate on in the points below.

2. I would also like to see a proper analysis for each pipeline (can be provided in supplemental information) describing comparisons to existing pipelines in terms of accuracy, resource usage, runtime stats, etc.

Minor points:

1. Introduction: "Such solutions are flexible and can help in pipeline implementation but do not provide robust standardized pipelines which are ready for production-scale analysis." In my experience, it's simply not true that WMS solutions are not suitable for production scale analysis. There are many examples of people doing exactly this, and moreover I've built several myself which are run multiple times every day without issue. In my experience they can work very reliably, and ability to tolerate and resume from errors is easy to code in. It is also unclear what the authors mean by standardisation in this context. I'd request that the authors either justify this point and provide concrete examples of exactly what the source of the perceived issues are, or remove this sentence.

2. Introduction: "These are useful for specific applications but can be challenging 25 to implement, difficult to modify or scale-up. They have also rarely been tested on multiple computing infrastructures." This seems too strong a statement. In some cases this might be true but there are many examples of robust pipelines that efficiently leverage data centre hardware.

3. Introduction: "GenPipes has been tested, benchmarked ...". It is not clear whether the "testing and

benchmarking" refers to the pipelines or the WMS itself. This should be clarified.
4. Schedulers: Ideally, GenPipes should offer the ability to implement scheduling via DRMMA which would increase the potential sites that could potentially run genpipe. For example, currently any data centres running Platform LSF could not use genpipe but via4 DRMMA this would be possible.
5. Job dependencies: I have reservations that the approach taken here is optimal. If I understand correctly, job dependencies are setup using the selected scheduler and all jobs, across steps, are launched at the same time. I suspect for very large pipelines containing many thousands jobs (not uncommon) this would put an undue burden on the scheduler and therefore would not scale very well. Could the authors elaborate on this point and highlight details such as what happens when a pipeline fails? Are existing jobs explicitly terminated? Or somehow left running and continue after the pipeline is resumed?
6. Configuration Files: "Configuration files, also referred to as "ini" files, are provided among the 20 arguments of the GenPipes command.". The authors should change the wording here. "Ini" is a legacy windows-based configuration file format. I'm not asking for the authors to change the configuration format used but it would be useful to have some justification for this unusual choice. Alternatives, like "yaml" for example allow for stricter and richer structuring and is therefore much easier to parse and in turn normally results in less buggy code.

Level of Interest

Please indicate how interesting you found the manuscript: Choose an item.

Quality of Written English

Please indicate the quality of language in the manuscript: Choose an item.

Declaration of Competing Interests

Please complete a declaration of competing interests, considering the following questions:

- Have you in the past five years received reimbursements, fees, funding, or salary from an organisation that may in any way gain or lose financially from the publication of this manuscript, either now or in the future?
- Do you hold any stocks or shares in an organisation that may in any way gain or lose financially from the publication of this manuscript, either now or in the future?
- Do you hold or are you currently applying for any patents relating to the content of the manuscript?
- Have you received reimbursements, fees, funding, or salary from an organization that holds or has applied for patents relating to the content of the manuscript?
- Do you have any other financial competing interests?
- Do you have any non-financial competing interests in relation to this paper?

If you can answer no to all of the above, write 'I declare that I have no competing interests' below. If your reply is yes to any, please give details below.

I declare that I have no competing interests

I agree to the open peer review policy of the journal. I understand that my name will be included on my report to the authors and, if the manuscript is accepted for publication, my named report including any attachments I upload will be posted on the website along with the authors' responses. I agree for my report to be made available under an Open Access Creative Commons CC-BY license (http://creativecommons.org/licenses/by/4.0/). I understand that any comments which I do not wish to be included in my named report can be included as confidential comments to the editors, which will not be published.

I agree to the open peer review policy of the journal

To further support our reviewers, we have joined with Publons, where you can gain additional credit to further highlight your hard work (see: https://publons.com/journal/530/gigascience). On publication of this paper, your review will be automatically added to Publons, you can then choose whether or not to claim your Publons credit. I understand this statement.

Yes Choose an item.
