# Supplementary material for: GenPipes: an open-source framework for distributed and scalable genomic analyses
Source: Gigascience. 2019 Jun 11;8(6):giz037. doi: 10.1093/gigascience/giz037 (PMC6559338; doi:10.1093/gigascience/giz037)
Supplement: giz037_Reviewer_2_Report_Original_Submission — Johannes KÃ¶ster -- 7/6/2018 Reviewed [file giz037_reviewer_2_report_original_submission.pdf]

Reviewer Report

Title: GenPipes: an open-source framework for distributed and scalable genomic analyses

Version: Original Submission **Date:** 7/6/2018

Reviewer name: Johannes KÃ¶ster

Reviewer Comments to Author:

The manscript presents GenPipes, a Python-based framework for defining and executing data analysis workflows.
GenPipes is based on a handful of Python classes that can be inherited and implemented to achieve a formal and executable description of a workflow in terms of steps.
During execution, steps are specialized to jobs that perform concrete operations on input files.
For me, the most important, and definitely valuable addition of this work is the comprehensive collection of well-tested workflows covering the most important applications of sequencing.
In general, I think this should be emphasized more, at the expense of removing some of the weaker aspects of the paper. I will outline this below.
Major Comments
* The manuscript argues that a major advantage of GenPipes is the rich collection of production-ready workflows that are delivered with the system. The list of workflows is indeed impressive, it should be mentioned though that both Snakemake and Nextflow also provide (community-maintained) collections of tested workflows, like github.com/snakemake-workflows, nf-core.github.io and sequana. I agree though that it might very well be that these are still less mature (except sequana), as they are probably newer.
* The manuscript claims that GenPipes supports cloud execution, but I cannot find a scheduler for this purpose in the list of schedulers on page 4. Also, the feature table says that cloud support is pending.
* On page 7, when describing deployment of software and reference information, it is unclear whether installation happens system wide (needing admin rights) or local. This should be clearly stated, since system-wide installation would be a major disadvantage compared to systems like Nextflow, Snakemake or CWL based WMSs. Moreover, it should be mentioned how those dependencies are updated, and in what sense such updates would affect previous runs, which could potentially loose reproducibility, if updates happen globally.
* In the discussion, it is mentioned that GenPipes is currently being reimplemented in WDL. It is a good choice to use one of the established, more feature-rich systems. However, then, large parts of this paper are in fact obsolete, as they will be replaced with WDL. The major contribution that remains after that step is the collection of workflows, which is totally fine, since this is a very valuable addition. I therefore suggest to put more focus on the workflows, and simply outline that they are currently implemented in GenPipes and soon will be avaialble in WDL. Morevoer, choice of tools, parameters and how the benchmarking was done (in a more concrete way instead of simply saying "we used GIAB") should be described in detail.
* Table 1 provides a feature comparison. As with every single feature comparison I have seen so far, it is

highly biased, showing only features that GenPipes itself provides. For example, GUI (as provided e.g. by Galaxy) and automatic reports are missing. Per-step/job software deployment and container support is missing. Config file validation is missing. Items are not sufficiently explained (e.g., what is meant with tracking, and in what sense is Nextflow not providing it). A popular system is completely missing from the table: Snakemake. Via nf-core and other projects, Nextflow and Snakemake provide several of the mentioned pipelines. Finally, I cannot actually find that table in reference [62], although the authors claim that it is a modified version of the table from that paper.
Minor Comments
* On page 2, when mentioning other WMSs, the authors should also mention Nextflow. Moreover, CWL and WDL are not WMSs, and should be listed separately as "declarative workflow description languages".
* On page 5, when relaunch features are mentioned, common functions of other systems like manual forcing or handling of missing files are not mentioned. Are these not available?
* On page 6, the description of input choice does not really make it clear how multiple input files or aggregation is handled. It would be beneficial to see examples for (a) a 1-in-1-out job, (b) an aggregating job, (c) a scattering job, (d) a mixed job (n-in-m-out).
* On page 8: "all workflows acceps a bam or fastq file as input". I guess they accept multiple bams or fastqs, right? Otherwise they could only be applied to a single sample at a time...

Level of Interest

Please indicate how interesting you found the manuscript: Choose an item.

Quality of Written English

Please indicate the quality of language in the manuscript: Choose an item.

Declaration of Competing Interests

Please complete a declaration of competing interests, considering the following questions:

- Have you in the past five years received reimbursements, fees, funding, or salary from an organisation that may in any way gain or lose financially from the publication of this manuscript, either now or in the future?
- Do you hold any stocks or shares in an organisation that may in any way gain or lose financially from the publication of this manuscript, either now or in the future?
- Do you hold or are you currently applying for any patents relating to the content of the manuscript?
- Have you received reimbursements, fees, funding, or salary from an organization that holds or has applied for patents relating to the content of the manuscript?
- Do you have any other financial competing interests?
- Do you have any non-financial competing interests in relation to this paper?

If you can answer no to all of the above, write 'I declare that I have no competing interests' below. If your reply is yes to any, please give details below.

I declare that I have no competing interests

I agree to the open peer review policy of the journal. I understand that my name will be included on my report to the authors and, if the manuscript is accepted for publication, my named report including any attachments I upload will be posted on the website along with the authors' responses. I agree for my report to be made available under an Open Access Creative Commons CC-BY license (http://creativecommons.org/licenses/by/4.0/). I understand that any comments which I do not wish to be included in my named report can be included as confidential comments to the editors, which will not be published.

I agree to the open peer review policy of the journal

To further support our reviewers, we have joined with Publons, where you can gain additional credit to further highlight your hard work (see: https://publons.com/journal/530/gigascience). On publication of this paper, your review will be automatically added to Publons, you can then choose whether or not to claim your Publons credit. I understand this statement.

Yes Choose an item.
