# Supplementary material for: GenPipes: an open-source framework for distributed and scalable genomic analyses
Source: Gigascience. 2019 Jun 11;8(6):giz037. doi: 10.1093/gigascience/giz037 (PMC6559338; doi:10.1093/gigascience/giz037)
Supplement: giz037_Reviewer_2_Report_Revision_1 — Johannes KÃ¶ster -- 11/20/2018 Reviewed [file giz037_reviewer_2_report_revision_1.pdf]

Reviewer Report

Title: GenPipes: an open-source framework for distributed and scalable genomic analyses

Version: Revision 1 **Date:** 11/20/2018

Reviewer name: Johannes KÃ¶ster

Reviewer Comments to Author:

The authors successfully address various of my and my colleagues requests. However, certain issues remain, which I will list in the following:
Major
* In the introduction, the authors say that frameworks like Galaxy can be inconvenient on large scale projects. Why is that? I think such a claim should be support by a detailed reasoning.
* When mentioning that WMSs rarely provide pre-built pipelines ready for production analysis, the authors should also mention that they nevertheless support development of such pipelines by the community of users, including linking out to examples like nf-core and github.com/snakemake-workflows.
* In my previous comment, I mentioned that the feature table is biased. While the authors added the columns suggested, these where only meant as examples. I would have thought that the authors take this as incentive to get a less biased view, which is arguably very hard. However, even when only taking the reviewer comments as a base, there are plenty of other columns which should go into the table. For example, the authors should add "DRMAA support", "status/progress monitoring" as a column. Moreover, the level of cloud support in GenPipes is quite different from what is offered by e.g. Nextflow and Snakemake. There, you have full Kubernetes support, in case of Snakemake even without the requirement of a shared filesystem. Maybe split the cloud column into "basic cloud support" and "kubernetes support".
* The installation mechanism for new software tools (outside of what is provided out of the box) (explained here: https://bitbucket.org/mugqic/genpipes/src/master/#markdown-header-modules), seems like manually redoing all the work that is already solved by package managers like conda or container engines like singularity. For example, Bioconda provides a library of over 4000 bioinformatics software packages which can be readily used from any WMS that supports conda, and Biocontainers provides the same for container based deployment (which lacks conda's ability to rapidly compose custom combinations of tools though). In order to make the comparison fair, the feature table should therefore contain two columns called "package-manager-integration" and "container-integration". For an example of what level of integration I am referring to, see https://snakemake.readthedocs.io/en/stable/snakefiles/deployment.html#integrated-package-management and https://www.nextflow.io/docs/latest/conda.html?highlight=conda.
* I am pleased to see that GenPipes indeed supports aggregation over many samples. What remains is the question whether the only entity to aggregate over are samples. If so, only over all samples or is it possible to express e.g. an arbitrary grouping of samples? Moreover, what about other properties, e.g. for scanning a parameter space? I suggest to somehow reflect the different ways of aggregation in the

feature table, maybe using the terms that I mentioned in my first review.
Minor
* Please mention in the caption of the feature table that community based workflows are not considered in the comparison. It might otherwise be that readers overlook this in the main text.
* Figure S1 contains a lot of typos, e.g. "reasdet", which I guess is supposed to be readset?

Level of Interest

Please indicate how interesting you found the manuscript: Choose an item.

Quality of Written English

Please indicate the quality of language in the manuscript: Choose an item.

Declaration of Competing Interests

Please complete a declaration of competing interests, considering the following questions:

- Have you in the past five years received reimbursements, fees, funding, or salary from an organisation that may in any way gain or lose financially from the publication of this manuscript, either now or in the future?
- Do you hold any stocks or shares in an organisation that may in any way gain or lose financially from the publication of this manuscript, either now or in the future?
- Do you hold or are you currently applying for any patents relating to the content of the manuscript?
- Have you received reimbursements, fees, funding, or salary from an organization that holds or has applied for patents relating to the content of the manuscript?
- Do you have any other financial competing interests?
- Do you have any non-financial competing interests in relation to this paper?

If you can answer no to all of the above, write 'I declare that I have no competing interests' below. If your reply is yes to any, please give details below.

I declare that I have no competing interests

I agree to the open peer review policy of the journal. I understand that my name will be included on my report to the authors and, if the manuscript is accepted for publication, my named report including any attachments I upload will be posted on the website along with the authors' responses. I agree for my report to be made available under an Open Access Creative Commons CC-BY license (http://creativecommons.org/licenses/by/4.0/). I understand that any comments which I do not wish to be included in my named report can be included as confidential comments to the editors, which will not be published.

I agree to the open peer review policy of the journal

To further support our reviewers, we have joined with Publons, where you can gain additional credit to further highlight your hard work (see: https://publons.com/journal/530/gigascience). On publication of this paper, your review will be automatically added to Publons, you can then choose whether or not to claim your Publons credit. I understand this statement.

Yes Choose an item.
