# Supplementary material for: GenPipes: an open-source framework for distributed and scalable genomic analyses
Source: Gigascience. 2019 Jun 11;8(6):giz037. doi: 10.1093/gigascience/giz037 (PMC6559338; doi:10.1093/gigascience/giz037)
Supplement: giz037_Reviewer_2_Report_Revision_2 — Johannes KÃ¶ster -- 1/30/2019 Reviewed [file giz037_reviewer_2_report_revision_2.pdf]

**Reviewer Report**

**Title: GenPipes: an open-source framework for distributed and scalable genomic analyses**

**Version: Revision 2** **Date:** 1/30/2019

**Reviewer name: Johannes KÃ¶ster**

**Reviewer Comments to Author:**

I thank the authors for addressing several of my concerns. Two issues remain:
# Major comments
* Regarding the feature table, the reasoning of the authors is acceptable (apart from my concern below), if the caption explicitly mentions that the table is "meant to provide the reader with an overview of the features of several tools in the field but not necessarily an exhaustive list".
* Regarding the answer "Bioconda offers a collection of packages and not an integrated system and can be quite heavy in memory requirements. Hence, we think that "package-manager-integration" is not necessarily an indication of the strength of the WMS. It is a specific choice, one that offers ease of installation but has its pitfalls as well. GenPipes does not use package managers by design. GenPipes manages its own libraries making sure there is no conflicting libraries in the process. For users who do not want to install GenPipes manually, we offer a Docker container that has also been tested with Singularity. We have updated the GenPipes' bitbucket documentation to highlight the availability of the GenPipes' Docker container.":
The way to provide the software stack for an analysis is very important for reproducibility and maintainability of a pipeline. Hence, this should definitely be a column of the feature table in any case. Certainly it is legitimate to decide against Conda or other package managerst, but in above argument I do not find any reason to hide this aspect from the user in the feature table. Why not adding a column about package manager support and let the reader decide whether they need/want this feature or not?

**Level of Interest**

Please indicate how interesting you found the manuscript: Choose an item.

**Quality of Written English**

Please indicate the quality of language in the manuscript: Choose an item.

**Declaration of Competing Interests**

Please complete a declaration of competing interests, considering the following questions:

- Have you in the past five years received reimbursements, fees, funding, or salary from an organisation that may in any way gain or lose financially from the publication of this manuscript, either now or in the future?
- Do you hold any stocks or shares in an organisation that may in any way gain or lose financially from the publication of this manuscript, either now or in the future?
- Do you hold or are you currently applying for any patents relating to the content of the manuscript?
- Have you received reimbursements, fees, funding, or salary from an organization that holds or has applied for patents relating to the content of the manuscript?
- Do you have any other financial competing interests?
- Do you have any non-financial competing interests in relation to this paper?

If you can answer no to all of the above, write 'I declare that I have no competing interests' below. If your reply is yes to any, please give details below.

I have no competing interests

I agree to the open peer review policy of the journal. I understand that my name will be included on my report to the authors and, if the manuscript is accepted for publication, my named report including any attachments I upload will be posted on the website along with the authors' responses. I agree for my report to be made available under an Open Access Creative Commons CC-BY license (http://creativecommons.org/licenses/by/4.0/). I understand that any comments which I do not wish to be included in my named report can be included as confidential comments to the editors, which will not be published.

I agree to the open peer review policy of the journal

To further support our reviewers, we have joined with Publons, where you can gain additional credit to further highlight your hard work (see: https://publons.com/journal/530/gigascience). On publication of this paper, your review will be automatically added to Publons, you can then choose whether or not to claim your Publons credit. I understand this statement.

Yes Choose an item.
